# Highly Pathogenic Avian Influenza A(H5N1) Virus in Poultry, Nigeria, 2015

**DOI:** 10.3201/eid2107.150421

**Published:** 2015-07

**Authors:** Isabella Monne, Clement Meseko, Tony Joannis, Ismaila Shittu, Mohammed Ahmed, Luca Tassoni, Alice Fusaro, Giovanni Cattoli

**Affiliations:** Istituto Zooprofilattico Sperimentale delle Venezie, Padova, Italy (I. Monne, L. Tassoni, A. Fusaro, G. Cattoli);; National Veterinary Research Institute, Vom, Nigeria (C. Meseko, T. Joannis, I. Shittu, M. Ahmed)

**Keywords:** highly pathogenic avian influenza virus A(H5N1), Nigeria, reassortment, viruses, influenza, poultry

**To the Editor:** In Nigeria, from February 2006 through July 2008, outbreaks of highly pathogenic avian influenza (HPAI) subtype H5N1 virus infection in poultry negatively affected animal and public health as well as the agricultural sector and trade. These outbreaks were caused by viruses belonging to genetic clades 2.2 and 2.2.1 (*1*). In January 2015, seven years after disappearance of the virus, clinical signs of HPAI (swollen head and wattles, hemorrhagic shank and feet) and increased mortality rates were observed among backyard poultry in Kano and in a live bird market in Lagos State, Nigeria. The virus was isolated from 2 samples independently collected from the poultry farm (parenchymatous tissues) and the market (tracheal swab), and H5 subtype virus was identified by reverse transcription PCR. The samples were adsorbed onto 2 Flinders Technology Associates cards (GE Healthcare Life Sciences, Little Chalfont, UK), which were sent to the World Organisation for Animal Health/Food and Agriculture Organization of the United Nations Reference Laboratory for Avian Influenza in Italy for subtype confirmation and genetic characterization. Influenza A(H5N1) virus was detected in both samples, and sequencing of the hemagglutinin (HA) gene showed that the viruses possessed the molecular markers for HPAI viruses with a multibasic amino acid cleavage site motif (PQRERRRKR*G).

The complete genome of the virus from backyard poultry was successfully sequenced from the genetic material extracted from the Flinders Technology Associates cards by using an Illumina MiSeq platform (*2*) and was submitted to the Global Initiative on Sharing All Influenza Data database (http://platform.gisaid.org/) under accession nos. EPI556504 and EPI567299–EPI567305. Maximum-likelihood trees were estimated for all 8 gene segments by using the best-fit general time reversible plus invariant sites plus gamma 4 model of nucleotide substitution with PhyML (*3*). The topology of the phylogenetic tree of the HA gene demonstrated that the H5N1 virus from Nigeria (A/chicken/Nigeria/15VIR339-2/2015) falls within genetic clade 2.3.2.1c (Figure, panel A). In particular, the HA gene sequence clustered with H5 viruses collected in China in 2013 and with an H5N1 virus (A/Alberta/01/2014) isolated from a Canada resident who had returned from China (similarity 99.3%–99.5%) (*4*).

The remaining 7 genes were closely related to genes of A/Alberta/01/2014(H5N1), although the 2 viruses differed by 32 aa (Technical Appendix). Just as for the virus from Canada (*4*), 7 of 8 gene segments of the virus from Nigeria clustered with HPAI A(H5N1) virus circulating in Vietnam and China, while the polymerase basic 2 gene segment (Figure, panel B) resulted from reassortment with viruses circulating in the same Asian countries but belonged to the H9N2 subtype. Differing from the strain from Canada (only 2 aa mutations compared with the 2.3.2.1c candidate vaccine strain; *5*), the strain from Nigeria possesses 6 aa differences: 3 in HA1 and 3 in HA2 (Technical Appendix). The effect of these mutations on the antigenic relatedness of these strains should be further explored.

**Figure:**
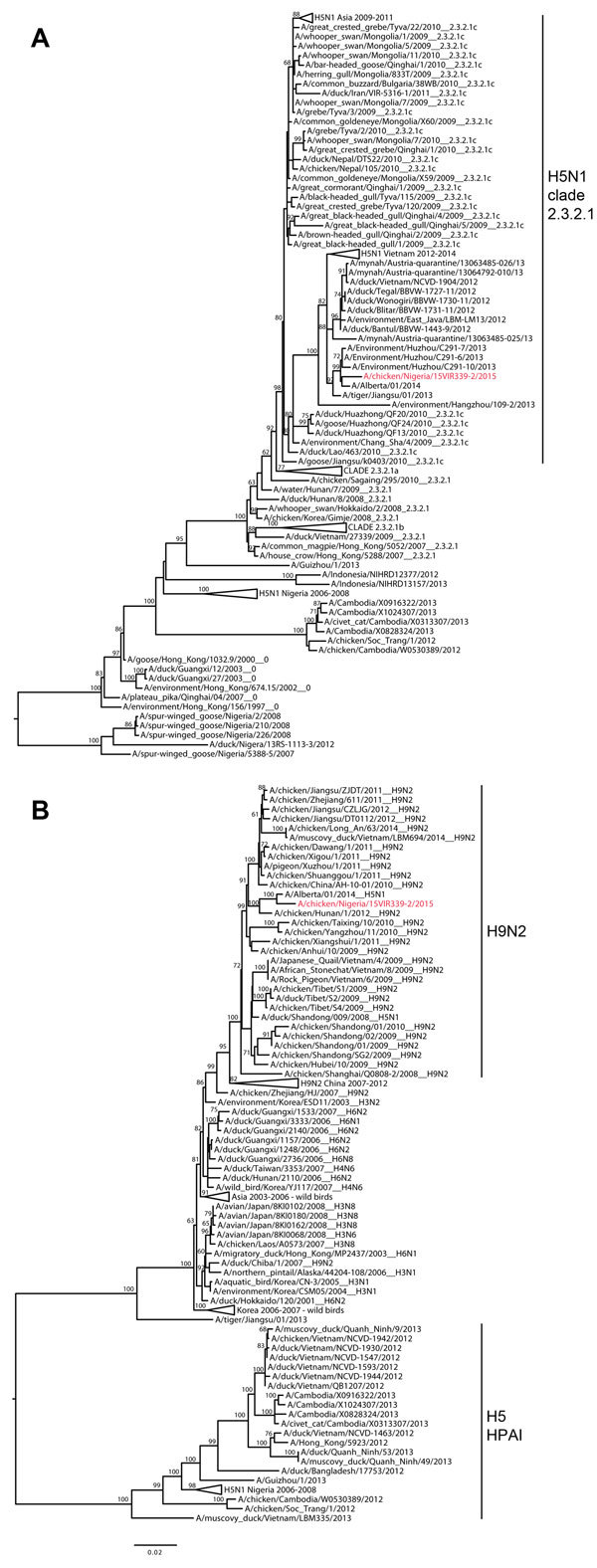
Maximum likelihood phylogenetic trees of the A) hemagglutinin and B) polymerase basic 2 gene segments of highly pathogenic avian influenza A(H5N1) virus from poultry in Nigeria, 2015 (in red). Bootstrap values (100 replicates) >60 are shown at the nodes. Scale bars indicate nucleotide substitutions per site. HPAI, highly pathogenic avian influenza.

Molecular characterization demonstrated that the polymerase basic 2 sequence contains glutamic acid at position 627, establishing the lack of a well-known mammalian adaptation motif (*6*). Mutations associated with increased virulence in mice have been observed in the nonstructural protein 1 (P42S, D87E, L98F, I101M, and the 80–84 deletion) and in the matrix 1 proteins (N30D, T215A). In addition, the substitutions D94N, S133A, S155N (H5 numbering) associated with increased binding to α-2,6 sialic acid have been identified in the HA protein. However, most of these substitutions are present in the H5N1 virus sequences from Asia included in our phylogenetic analyses, suggesting that they may be common among the HPAI H5 virus subtype. Mutations associated with resistance to antiviral drugs have not been detected (*7*).

The results obtained from whole-genome analysis provide evidence that a novel clade of the A(H5N1) virus, specifically clade 2.3.2.1c, has reached Nigeria. Although ascertaining how and exactly when this has happened is difficult, it seems most likely that the virus entered the country in December 2014, as evidenced by unverified accounts of increased poultry deaths in some live bird markets in Lagos, after the birds had been moved from the north (Kano) to the south during the festive season. The identification of genetic clustering between the strains from Nigeria analyzed here and the HPAI A(H5N1) viruses originally identified in Asia suggests an unknown epidemiologic link between these regions, probably associated with human activities, migratory bird movements, or both.

Considering that this virus is an intersubtype reassortant and has already caused infection in humans, we believe that complete characterization of the strain in terms of virulence and host range is of high priority. Furthermore, because the reemergence of subtype H5N1 virus was followed by epidemiologic amplification (≈265 outbreaks in 18 states as of February 2015; T. Joannis, pers. comm., 2015) for which virus genetic characterization is not yet available, local veterinary and public health services and international organizations should take necessary measures to identify critical control points and stop circulation of this virus.

**Technical Appendix.** Amino acid comparison of avian influenza viruses and sequences from the Global Initiative on Sharing All Influenza Data database.
