# Supplementary material for: Highly Pathogenic Avian Influenza A(H5N1) Virus in Poultry, Nigeria, 2015
Source: Emerg Infect Dis. 2015 Jul;21(7):1275–7. doi: 10.3201/eid2107.150421 (PMC4480409; doi:10.3201/eid2107.150421)
Supplement: Supplementary file 1 — Technical Appendix. Amino acid comparison of avian influenza viruses and sequences from the Global Initiative on Sharing All Influenza Data database. [file 15-0421-Techapp-s1.pdf]

Article DOI: http://dx.doi.org/10.3201/eid2107.150421

Highly Pathogenic Avian Influenza A(H5N1) Virus in Poultry, Nigeria, 2015

Technical Appendix

Technical Appendix Table 1. Amino acid comparison among A/chicken/Nigeria/15VIR339-2/2015, A/Alberta/01/2014 and the 2.3.2.1c vaccine candidate A/duck/Vietnam/NCVD-1584/2012*

Protein	Position	H5N1 Nigeria	H5N1 Alberta	2.3.2.1c vaccine candidate
HA1	68	G	D	D
	189	K	K	R
	221	G	R	G
	235	T	P	P
HA2	48	I	V	V
	68	K	R	R
	183	V	I	I
NA	267	E	G	-
	398	M	I	-
PA	319	D	E	-
	369	V	A	-
	474	S	C	-
NS1	59	S	R	-
	72	G	E	-
	124	L	I	-
	134	D	N	-
	166	S	G	-
NS2	36	E	G	-
PB1	179	I	M	-
	384	S	L	-
	744	I	M	-
	758	Q	*	-
PB1-F2	34	S	N	-
	45	T	I	-
	58	W	*	-
PB2	187	K	R	-
	196	C	S	-
	292	V	I	-
	464	M	L	-
	495	A	V	-
	598	T	M	-
	660	R	K	-
	677	G	E	-

*World Health Organization. Antigenic and genetic characteristics of zoonotic influenza viruses and development of candidate vaccine viruses for pandemic preparedness (http://www.who.int/influenza/vaccines/virus/characteristics_virus_vaccines/en/).

Technical Appendix Table 2. Sequences from GISAID's EpiFlu™ Database on which this research is based*

Segment ID	Segment	Country	Collection date	Isolate name	Originating Lab	Submitting Laboratory	Authors
EPI500778	PB2	Canada	2014-01-03	A/Alberta/01/2014	Provincial Laboratory of Public Health for Southern Alberta	Public Health Agency of Canada (PHAC)	Li, Yan; Bastien, Nathalie; Fonseca, Kevin; Tipples, Graham; Pabbaraju, Kanti; Tellier, Raymond; Wong, Sallene; Tang, Julian W.; Drews, Steven J.
EPI466327	PB2	China	2012-03-24	A/chicken/Jiangsu/CZLJG/2012	Yangzhou University	Chinese Academy of Sciences	Daxin, P
EPI466359	PB2	China	2012-02-12	A/chicken/Jiangsu/DT0112/2012	Yangzhou University	Chinese Academy of Sciences	Daxin, P
EPI466536	PB2	China	2012-09-24	A/chicken/Jiangsu/GY64/2012	Yangzhou University	Chinese Academy of Sciences	Daxin, P
EPI466235	PB2	China	2012-03-24	A/chicken/Jiangsu/WJHDL/2012	Yangzhou University	Chinese Academy of Sciences	Daxin, P
EPI466520	PB2	China	2012-11-09	A/chicken/Jiangsu/YZ4/2012	Yangzhou University	Chinese Academy of Sciences	Daxin, P
EPI466311	PB2	China	2011-05-21	A/chicken/Jiangsu/ZJDT/2011	Yangzhou University	Chinese Academy of Sciences	Daxin, P
EPI425173	PB2	Vietnam	2012-09-01	A/chicken/Vietnam/NCVD-1942/2012	National Centre of Veterinary Diagnostics	Centers for Disease Control and Prevention	
EPI425597	PB2	Vietnam	2012-09-21	A/duck/Vietnam/NCVD-1463/2012	National Centre of Veterinary Diagnostics	Centers for Disease Control and Prevention	
EPI425920	PB2	Vietnam	2012-07-16	A/duck/Vietnam/NCVD-1547/2012	National Centre of Veterinary Diagnostics	Centers for Disease Control and Prevention	
EPI425677	PB2	Vietnam	2012-07-15	A/duck/Vietnam/NCVD-1593/2012	National Centre of Veterinary Diagnostics	Centers for Disease Control and Prevention	
EPI425621	PB2	Vietnam	2012-08-18	A/duck/Vietnam/NCVD-1930/2012	National Centre of Veterinary Diagnostics	Centers for Disease Control and Prevention	
EPI425181	PB2	Vietnam	2012-09-09	A/duck/Vietnam/NCVD-1944/2012	National Centre of Veterinary Diagnostics	Centers for Disease Control and Prevention	
EPI468343	PB2	China	2013-04-12	A/environment/Hangzhou/109-2/2013	Hangzhou Center for Disease Control and Prevention	Hangzhou Center for Disease Control and Prevention	Li, J; Jin, T; Yu, XF; Pu, XY; Pan, JC
EPI423876	PB2	China	2013-02-08	A/Guizhou/1/2013	WHO Chinese National Influenza Center	China National Influenza Centre	
EPI375501	PB2	China	2012-05-28	A/Hong Kong/5923/2012	Public Health Laboratory Services Branch, Centre for Health Protection	Public Health Laboratory Services Branch, Centre for Health Protection	Mak,G.C.; Cheng,P.K.C.; Lo,J.Y.C.
EPI375432	HA	China	2012-05-28	A/Hong Kong/5923/2012	Public Health Laboratory Services Branch,	Public Health Laboratory Services Branch, Centre for	Mak,G.C.; Cheng,P.K.C.; Lo,J.Y.C.

Segment ID	Segment	Country	Collection date	Isolate name	Originating Lab	Submitting Laboratory	Authors
					Centre for Health Protection	Health Protection	
EPI407283	HA	Nepal	2012-10-16	A/chicken/Nepal/PT-16/12	Central Veterinary Laboratory	Animal Health and Veterinary Laboratories Agency (AHVLA)	Puranik, A; Hanna, A; Essen, S; Focosi-Snyman, R; Manvell, R; Bahadur Singh, D; Chapagain, S; Manandhar, S; Bahadur Air, T; Bahadur Kunwar, B; Reid, S
EPI420386	HA	China	2013-02-08	A/Guizhou/1/2013	WHO Chinese National Influenza Center	China National Influenza Centre	
EPI424704	HA	Vietnam	2012-08-20	A/duck/Vietnam/ NCVD-1869/2012	National Centre of Veterinary Diagnostics	Centers for Disease Control and Prevention	
EPI424720	HA	Vietnam	2012-08-23	A/duck/Vietnam/ NCVD-1897/2012	National Centre of Veterinary Diagnostics	Centers for Disease Control and Prevention	
EPI424728	HA	Vietnam	2012-08-27	A/duck/Vietnam/ NCVD-1898/2012	National Centre of Veterinary Diagnostics	Centers for Disease Control and Prevention	
EPI425184	HA	Vietnam	2012-09-09	A/duck/Vietnam/N CVD-1944/2012	National Centre of Veterinary Diagnostics	Centers for Disease Control and Prevention	
EPI425600	HA	Vietnam	2012-09-21	A/duck/Vietnam/ NCVD-1463/2012	National Centre of Veterinary Diagnostics	Centers for Disease Control and Prevention	
EPI442759	HA	Indonesia	2012-06-29	A/Indonesia/ NIHRD12377/2012	National Institute of Health Research and Development	National Institute of Health Research and Development	Pawestri, HA; Ikawati, HD; Setiawaty, V
EPI454493	HA	China	2013-04-12	A/environment/ Hangzhou/109-2/2013	Hangzhou Center for Disease Control and Prevention	Hangzhou Center for Disease Control and Prevention	Li, J; Jin, T; Yu, XF; Pu, XY; Pan, JC
EPI463648	HA	Indonesia	2013-06-18	A/Indonesia/ NIHRD13157/2013	National Institute of Health Research and Development	National Institute of Health Research and Development	HA, Pawestri; AA, Nugraha; V, Setiawaty
EPI475764	HA	Nepal	2013-04-21	A/chicken/Nepal/T-272/13	Central Veterinary Laboratory	Animal Health and Veterinary Laboratories Agency (AHVLA)	Collins, S; Hanna, A; Essen, S; Focosi-Snyman, R; Manvell, R; Jha, VC; Chapagain, S; Koirala, P; Air, TB; Reid, S
EPI448063	HA	Bangladesh	2011-02-15	A/crow/Bangladesh/ 1061/2011	Institute of Epidemiology Disease Control and Research (IEDCR) & Bangladesh National Influenza Centre (NIC)	Centers for Disease Control and Prevention	Gerloff, Nancy; Simpson, Natosha; Poh, Mee;Davis, Todd
EPI353379	HA	Bangladesh	2011-07-17	A/waterfowl/Bangladesh/31935/2011	Institute of Epidemiology Disease Control and Research	Centers for Disease Control and Prevention	Gerloff, Nancy; Simpson, Natosha; Poh, Mee;Davis, Todd

Segment ID	Segment	Country	Collection date	Isolate name	Originating Lab	Submitting Laboratory	Authors
					(IEDCR) & Bangladesh National Influenza Centre (NIC)		
EPI448263	HA	Bangladesh	2012-02-28	A/environment/Bangladesh/1019-G/2012	Institute of Epidemiology Disease Control and Research (IEDCR) & Bangladesh National Influenza Centre (NIC)	Centers for Disease Control and Prevention	Gerloff, Nancy; Simpson, Natosha; Poh, Mee; Davis, Todd
EPI448271	HA	Bangladesh	2012-01-08	A/chicken/Bangladesh/42010/2012	Institute of Epidemiology Disease Control and Research (IEDCR) & Bangladesh National Influenza Centre (NIC)	Centers for Disease Control and Prevention	Gerloff, Nancy; Simpson, Natosha; Poh, Mee; Davis, Todd
EPI448279	HA	Bangladesh	2012-02-20	A/duck/Bangladesh/32077/2012	Institute of Epidemiology Disease Control and Research (IEDCR) & Bangladesh National Influenza Centre (NIC)	Centers for Disease Control and Prevention	Gerloff, Nancy; Simpson, Natosha; Poh, Mee; Davis, Todd
EPI425200	HA	Vietnam	2011-11-22	A/duck/Vietnam/NCVD129-7/2011	National Centre of Veterinary Diagnostics	Centers for Disease Control and Prevention	
EPI425232	HA	Vietnam	2011-12-30	A/duck/Vietnam/NCVD-1160/2011	National Centre of Veterinary Diagnostics	Centers for Disease Control and Prevention	
EPI375432	HA	China	2012-05-28	A/Hong Kong/5923/2012	Public Health Laboratory Services Branch, Centre for Health Protection	Public Health Laboratory Services Branch, Centre for Health Protection	Mak, G.C.; Cheng, P.K.C.; Lo, J.Y.C.
EPI330995	HA	Vietnam	2011-01	A/duck/Vietnam/NCVD-672/2011	National Centre of Veterinary Diagnostics	Centers for Disease Control and Prevention	Davis, Todd; Rivailler, Pierre; Nguyen, Tung
EPI267032	HA	China	2010-06-01	A/Hubei/1/2010		WHO Chinese National Influenza Center	Yu Lan, Wei Wang, Shumei Zou, Zi Li, Leying Wen, Xiaodan Li, Libo Dong, Dexin Li, Yuelong Shu
EPI425312	HA	Vietnam	2012-08-30	A/duck/Vietnam/NCVD-1904/2012	National Centre of Veterinary Diagnostics	Centers for Disease Control and Prevention	
EPI462795	HA	Austria	2013-06-12	A/mynah/Austria-quarantine/13064792-010/13	Institute for Veterinary Disease	Animal Health and Veterinary	Collins, S; Hanna, A; Essen, S; Focosi-Snyman, R; Manvell, R;

Segment ID	Segment	Country	Collection date	Isolate name	Originating Lab	Submitting Laboratory	Authors
					Control Moedling, Austrian Agency for Health and Food Safety	Laboratories Agency (AHVLA)	Wodak, E; Revilla-Fernandez, S; Bago, Z; Schmoll, F; Reid, S
EPI462793	HA	Austria	2013-06-07	A/mynah/Austria-quarantine/13063485-026/13	Institute for Veterinary Disease Control Moedling, Austrian Agency for Health and Food Safety	Animal Health and Veterinary Laboratories Agency (AHVLA)	Collins, S; Hanna, A; Essen, S; Focosi-Snyman, R; Manvell, R; Wodak, E; Revilla-Fernandez, S; Bag?, Z; Schmoll, F; Reid, S
EPI462791	HA	Austria	2013-06-07	A/mynah/Austria-quarantine/13063485-025/13	Institute for Veterinary Disease Control Moedling, Austrian Agency for Health and Food Safety	Animal Health and Veterinary Laboratories Agency (AHVLA)	Collins, S; Hanna, A; Essen, S; Focosi-Snyman, R; Manvell, R; Wodak, E; Revilla-Fernandez, S; Bag?, Z; Schmoll, F; Reid, S
EPI464565	HA	China	2013-04-16	A/Environment/Huzhou/C291-7/2013	Huzhou Center for Disease Control and Prevention	Jiangsu University	Han, J. ; Wang, L. ; Liu, J. ; Jin, M. ; Zhang, C. ; Lan, K
EPI464564	HA	China	2013-04-16	A/Environment/Huzhou/C291-6/2013	Huzhou Center for Disease Control and Prevention	Jiangsu University	Han, J. ; Wang, L. ; Liu, J. ; Jin, M. ; Zhang, C. ; Lan, K
EPI464558	HA	China	2013-04-16	A/Environment/Huzhou/C291-10/2013	Huzhou Center for Disease Control and Prevention	Jiangsu University	Han, J. ; Wang, L. ; Liu, J. ; Jin, M. ; Zhang, C. ; Lan, K
EPI500771	HA	Canada	2014-01-03	A/Alberta/01/2014	Provincial Laboratory of Public Health for Southern Alberta	Public Health Agency of Canada (PHAC)	Li, Yan; Bastien, Nathalie; Fonseca, Kevin; Tipples, Graham; Pabbaraju, Kanti; Tellier, Raymond; Wong, Sallene; Tang, Julian W.; Drews, Steven J.

*We gratefully acknowledge the authors, originating and submitting laboratories of sequences from GISAID's EpiFlu Database, on which this research is based All Submitters of data may be contacted directly via the GISAID website www.gisaid.org
